# Prevention of Alcohol, Tobacco, and Illicit Drug Use Among Youth: A Scoping Review of European School-Based Programs with Insights on Mental Health

**DOI:** 10.3390/ijerph22101569

**Published:** 2025-10-15

**Authors:** Ahmed Abdelrahman, Luz Bernad, Fiona Harris, Elodie Rezzonico, Antoine Flahault, Jennifer Hasselgard-Rowe

**Affiliations:** 1 Global Studies Institute, University of Geneva, 1205 Geneva, Switzerland; ahmed.abdelrahman@etu.unige.ch (A.A.); luz.bernad@etu.unige.ch (L.B.); elodie.rezzonico@etu.unige.ch (E.R.); 2 Faculty of Medicine, Institute of Global Health, University of Geneva, 1211 Geneva, Switzerland; antoine.flahault@unige.ch (A.F.); jennifer.hasselgard-rowe@unige.ch (J.H.-R.)

**Keywords:** prevention, school-based interventions, alcohol, drugs, Europe, Preventure, Unplugged, IPSYcare, adolescents

## Abstract

Alcohol, tobacco, and drug misuse continue to rise globally, with adolescents at particular risk. In response, school-based prevention programs have been widely implemented, yet their efficacy and long-term impact remain under-discussed. This scoping review synthesised evidence on the effectiveness of three commonly used programs (Preventure, Unplugged, and IPSYcare) in Europe. A search of four databases (PubMed, Embase, PsycInfo, and Web of Science) identified 21 peer-reviewed articles published between 2008 and 2023, spanning 12 European countries. Unplugged was most frequently evaluated (10 studies), followed by Preventure (6 studies) and IPSYcare (5 studies). Findings showed that Preventure yielded mixed outcomes, delaying binge drinking and reducing substance use among high-risk groups but with limited generalisability. Unplugged was associated with reductions in cannabis use and heavy drinking at 15 months post-intervention. IPSYcare demonstrated longer-term benefits, including improved school connectedness and reductions in alcohol and tobacco use. Results suggest that while standardised programs such as Unplugged enable scalability, contextual adaptations may enhance effectiveness, and tailored approaches are valuable for high-risk populations. Overall, the programs show potential, but variability indicate the need for further longitudinal and qualitative research in order to improve program delivery and sustain long-term impacts.

## 1. Introduction

Alcohol and drug misuse are increasing health concerns worldwide, with significant impacts on health, social systems, and economic productivity [1,2]. Classified as psychoactive substances, alcohol, tobacco, and other drugs alter brain function and behaviours, promoting addiction and causing severe long-term conditions, including chronic diseases [3]. Globally, alcohol alone is responsible for 3.3 million deaths, accounting for 5.1% of the total burden of disease. The impact of alcohol on mental health is markedly higher in European countries, where it contributes to 1% of disability-adjusted life years (DALYs) compared to 0.49% globally [3]. In addition, drug use prevalence among young people varies greatly between regions. In Europe it can reach 32.5% prevalence for some substances, compared to 5% in Latin America and 9.5% in North America [4].

Young adults and teenagers are particularly vulnerable to substance misuse due to changing social roles, greater independence from parents and increased stress and anxiety. Adolescents and young adults might have a strong understanding of the health and social consequences of substance abuse; however, this does not translate into the behaviours required to protect their health. The set of skills and knowledge required for self-care develop progressively during adolescence and does not reach its full maturity until early adulthood [1]. Consequently, most people who abuse substances initiate these behaviours during their schooling, as this developmental process is not yet completed [2,4].

Schools provide structured environments for the integration of substance prevention programs in the curricula, offering a systemic and efficient way to reach large numbers of young adults [4]. Therefore, school-based intervention programs have become the primary approach to address alcohol and substance misuse, with their implementation expanding during the last few decades [2,4,5]. Such strategies aim to delay alcohol and drug initiation while preventing progression to addiction by strengthening self-care skills and protective behaviours [1,2,4]. Europe has been at the forefront of alcohol and substance prevention, with numerous programs developed and implemented, among which Preventure, Unplugged, and IPSYcare (Information + Psychological competence = Protection) are among the most widely adopted [4]. While Europe focuses on more universally targeted approaches, which target all students regardless of their substance use level and risk behaviours, North America often emphasises personality behaviours and traits which are associated with increased alcohol and substance abuse risk. Consequently, Preventure-based programs are particularly common in this region, which are developed around the substance use risk profile scale [5,6].

### 1.1. Program Characteristics and Delivery

#### 1.1.1. Preventure

Preventure programs are based on cognitive behavioural strategies and personality profiles and provide two 90 min group sessions under the supervision of trained counsellors to address vulnerabilities, foster resilience, and make informed decisions regarding substance use. Sessions focus on building coping strategies rather than directly addressing substance use and are tailored to the four personality profiles (AS, NT, IMP, and SS (AS: Anxiety Sensitivity; NT: Negative Thinking; IMP: Impulsivity; SS: Sensation Seeking)) as identified by the Substance Use Risk Profile Scale (SURPS) [6]. The first session provides personality traits knowledge and the maladaptive behaviours associated, while the second session focuses on identifying personality-specific thoughts that lead to problematic behaviours [6].

#### 1.1.2. Unplugged

Based on the Comprehensive Social Influence Model, Unplugged, delivered via an interactive set of 12 one-hour weekly lessons, is designed to address experimental and ongoing use of tobacco, alcohol, and illicit drugs such as cannabis. The lessons aimed to increase knowledge, build social skills, and foster interpersonal skills through the application of refusal skills, critical thinking, decision-making, and goal setting [7]. Teachers were trained using a standardised 3-day training course and provided with a handbook to facilitate delivery. Data on the frequency of substance use in the past 30 days was collected through self-reported confidential questionnaires [7]. The curriculum and questionnaires were translated into the local language, but no other modifications to content were made.

#### 1.1.3. IPSYcare

IPSYcare is a universal school-based prevention program that is grounded in the World Health Organization’s (WHO) life skills education model and psychological theories of adolescent development [8,9,10,11,12]. The program is delivered to all students in grades 5–7 (ages 11–13) over a three-year period, regardless of prior risk or substance use and aims to strengthen intrapersonal and interpersonal competencies such as communication, stress coping, and problem solving, alongside substance-specific skills including refusal strategies [8,9,10]. The curriculum also incorporates modules designed to promote school bonding and positive attitudes towards learning [10]. By targeting all students in regular class settings, the program seeks to strengthen protective factors whilst reducing risk factors for substance use in early adolescence [8,9,10].

The program is implemented by classroom teachers, who receive a one-day training, and is embedded in the regular school curriculum followed by students. Teaching is conducted through interactive methods such as role-play, group discussions, and reflection exercises [8,9]. IPSYcare consists of a basic module in grade 5 (consisting of 15 lessons, of which 10 last for approximately 90 min and the remaining 5 for 45 min), followed by two booster modules in grades 6 and 7 (7 lessons each, of which 4 last for 90 min and the remaining 3 for 45 min) totalling approximately 33 h [9]. Self-reported questionnaires from teachers and students after each session are used to report the data. Students are assessed on their alcohol and tobacco consumption frequency and likelihood of experimentation with substances [8].

### 1.2. Objective

Although these programs have shown potential, their efficacy and long-term impact remain discussed. Issues, including variations in implementation, cultural differences, and resource constraints, hinder the ability to draw definite conclusions [2,7]. Moreover, the outdated nature of many of these studies, along with inconsistencies in follow-up methodologies, further undermines the studies’ results’ reliability [13].

Given Europe’s high prevalence of alcohol and substance abuse, as well as their leading role in the development and implementation of school-based intervention programs, it is crucial to synthesise the evidence specific to this region. A comprehensive and contextually informed review of the existing evidence is required to understand how these programs function and their outcomes across the European context.

This scoping review therefore aims to map and synthesise the existing literature on the school-based prevention programs Preventure, Unplugged, and IPSYcare in Europe. Specifically, it aims to provide an overview of the characteristics, implementation strategies and outcomes across these programs. It also aims to assess their effectiveness in preventing or reducing alcohol, tobacco and drug use while improving mental health outcomes among European adolescents and to identify gaps and areas for future research.

## 2. Materials and Methods

The team searched PubMed, Embase, PsychInfo, and Web of Science databases. The search terms included: “Unplugged OR Preventure OR IPSY OR IPSYcare” AND “depression OR anxiety OR mental health OR psychological disorders” AND “alcohol OR drug* OR cannabis” for results published in each database from inception to 11 November 2024.

Relevant aspects of the studies were compiled in an Excel file and duplicates were removed before the researchers worked in independent pairs to analyse the titles and abstracts to assess whether they met the following inclusion criteria: (a) Unplugged, Preventure, or IPSYcare school-based prevention program, (b) conducted in Europe, (c) addressing tobacco and/or alcohol and/or drug use, and (d) written in English, Spanish, or French. Exclusion criteria included: (a) books, book chapters, and congresses reports, and (b) no publishable results, defined here as records lacking sufficient methodological or outcome information for data extraction, e.g., abstracts without full text availability, reports that summarised preliminary results without statistical or outcome data, and grey literature that lacked clear analytic information. Discrepancies were resolved through critical discussion between the whole team until consensus was achieved.

Data from the selected studies was extracted through a specifically designed data extraction form developed for this review in accordance with the Preferred Reporting Items for Systematic Reviews and Meta-analysis extension for scoping reviews (PRISMA-ScR) [14]. This review was not registered. Two reviewers independently extracted data from each article. Appendix A Table A1 contains detailed data extraction results including the program reviewed, country, gender and age of participants, study outcomes, and additional findings. In addition, researchers skimmed the reference list of each paper to find additional papers that may have been missed during the initial search. The selected studies were grouped according to program and analysed using descriptive analysis and narrative synthesis.

## 3. Results

**Figure 1:**
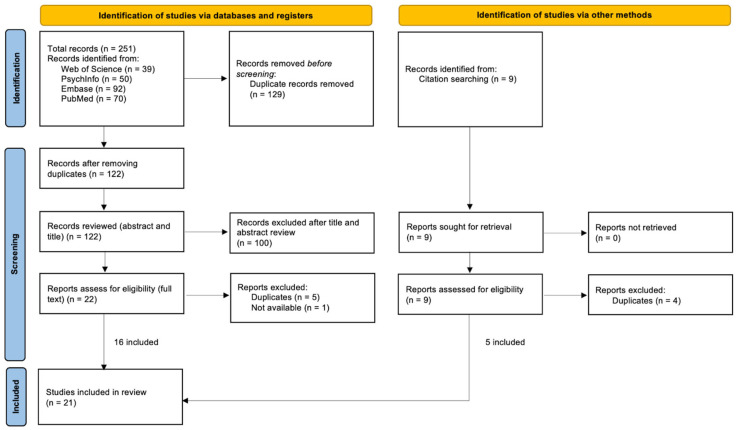
PRISMA flow diagram.

The initial search yielded 251 results: 39 from Web of Science, 50 from PsychInfo, 92 from Embase, and 70 from PubMed. After eliminating duplicates, 122 articles were selected for title and abstract review. 100 articles were excluded for not meeting inclusion criteria. The full text of the remaining 22 articles was reviewed; 5 duplicate articles were removed and 1 was unavailable. A total of 9 additional articles were identified by citation searching, 5 of which were added for a total of 21 articles. Figure 1 shows the review process.

### 3.1. Overall Characteristics

The selected studies were published between 2008 and 2023. Unplugged (*n* = 10, 47.6%) was the most frequently evaluated program, followed by Preventure (*n* = 6, 28.6%) and IPSYcare (*n* = 5, 23.8%). Studies spanned across 12 European countries, with Germany (*n* = 6, 31.6%), Spain (*n* = 5, 26.3%), and the Czech Republic (*n* = 4, 21.1%) as the most common. Most research centred on Western and Central Europe, with Southern Europe included within multi-country studies.

### 3.2. Program Implementation and Study Design

#### 3.2.1. Preventure

Six controlled randomised trials conducted between 2013 and 2019 were included [15,16,17,18,19,20]. Three were conducted in the United Kingdom [15,16,17], and three in the Netherlands [18,19,20]. Sample sizes ranged from 2643 to 699 participants, with mean reported ages varying between 13.7 and 15 years old. Studies conducted in the United Kingdom included follow-ups conducted every six months over a two-year period; those in the Netherlands included follow-ups at two, six and twelve-months post-intervention. Both used online questionnaires.

Participants who benefited from the intervention all scored at least one standard deviation (SD) above the sample mean on the SURPS and therefore belonged to the high-risk group [15,16,17,18,19,20]. However, only one study included the low-risk students in their follow-up assessment [15]. If a student had a high score (≥1 SD) on more than one SURPS subscale, they were assigned to the session corresponding to the personality trait with the highest z-score [16,17,19,20].

While all studies had the aim to evaluate the effectiveness of the Preventure program on alcohol misuse, some had additional objectives. Precisely, one study focused on the influence of two contextual risk factors, namely socioeconomic status and peer victimisation, on the effectiveness of the program among high-risk students [16]. One study explored the potential herd effect of the intervention on low-risk peers [15]. One study focused on gender and level of education [19]. The effectiveness of the program on a selection of mental health outcomes was tested in two studies [17,20].

#### 3.2.2. Unplugged

Ten Unplugged studies were included [21,22,23,24,25,26,27,28,29,30]. Six studies used data from the 2004 Europe Drug Abuse Prevention trial (EU-Dap) conducted in 7 countries (Austria, Belgium, Germany, Greece, Italy, Spain, and Sweden) and involved 7079 students aged 12 to 14 years old [22,23,24,25,28,29]. Three studies focused on an Unplugged trial conducted in the Czech Republic in 2007 which involved 1874 students aged 12 to 14 years old [26,27,30]. One study was conducted in Slovakia over six months which involved 425 students with a mean age of 13.5 years [21]. Two systematic reviews examined multiple school-based prevention programs, in addition to Unplugged programs [22,29].

Four studies examined the use of alcohol, tobacco, and cannabis [24,25,27,28]. One examined alcohol and tobacco [21]. Three studies focused solely on alcohol [23], and two on tobacco [26,30]. Follow-up to the EU-Dap trial was conducted at three months in three studies [24,25,28], and at 18 months for two studies [23,25]; at six months for the Slovakian trial [21], and at one, three, 12, 15, and 24 months in the Czech studies [26,27,30]. Retention rates for the EU-Dap studies were 93.3% at three months and 81.3% at eighteen months [23], 78.4% in Slovakia [21], and 93.5% in the Czech Republic [30].

#### 3.2.3. IPSYcare

Five IPSYcare articles were included [8,9,10,11,12]. One early pilot study tested the feasibility of peer- vs. teacher-led delivery formats using a pre- and post-test design on 105 students between grades 5 and 7 in Thuringia, Germany [11]. It examined implementation quality, participant satisfaction, and short-term changes in alcohol and tobacco use, providing a basis in order to refine the program implementation before large-scale rollout [11].

The remaining four studies [8,9,10] were based on data from a 2003 quasi-experimental longitudinal study that was conducted in the same location as the pilot study and involved 1675 students across 44 schools (23 that included the IPSYcare program into the curricula and 21 schools that followed the regular German schooling program). Data were collected at four intervals—pre-test (autumn 2003), post-test (spring 2004), and two annual follow-ups (one in grades 6 and 7)—with one study extending into grades 8 and 9 to assess post-program sustainability and any longer-term program effects [8]. One study also made reference to results from replication studies conducted in Austria and Italy to assess the program’s applicability across other European contexts [9].

While all studies looked at IPSYcare’s effectiveness in delaying or reducing alcohol, tobacco and/or substance use, some studies also examined distinct but complementary aspects of the program’s preventive framework. One study investigated whether school bonding mediated reductions in alcohol use [10]. Another analysis made use of a growth mixture model to identify distinct developmental patterns of drinking. This technique grouped adolescents according to similar patterns in alcohol use and how this progressed over three years. Through this method a normative group (comprising about 80% of students, meaning they either did not or only initiated drinking at a later age) and a problematic group (about 20%, who began drinking early and more frequently) were identified [12]. Another study looked at whether the program had any effect on students attending different school tracks (academic vs. vocational) as well as for boys and girls [8].

Furthermore, program acceptance was also high, with approximately 80% of students reporting they would participate again, with teachers successfully delivering an estimated 80–90% of the IPSYcare manual content [9].

### 3.3. Program Effectiveness

#### 3.3.1. Preventure

One study found no significant effects on alcohol use and binge drinking twelve months post-intervention [18]. However, it did report a preventive effect on binge drinking frequency at twelve months post-intervention, suggesting that the intervention may delay drinking behaviours. Another study reported that scoring high on sensation-seeking behaviour delayed binge drinking and frequency during the twelve-month follow-up [19]. Two studies reported reduced alcohol consumption in individuals with anxiety twelve months post intervention [15,19]. Further, high-risk groups (anxiety, negative thinking, impulsivity and hopelessness) exhibited a reduction in the frequency of drinking and binge drinking [15]. The program also appeared effective in reducing substance misuse in a two-year period in youth at particularly high risk for addiction and mental health problems [17]; however, results are mixed, as one study reported positive effects on anxiety reduction within the anxiety-sensitive group (AS) but a negative effect on depression within the negative-thinking group (NT) at twelve months post-intervention [20]. Psychiatric symptoms of hyperactivity/impulsivity and conduct problems had a moderating effect on the intervention. Specifically, adolescents with high levels of these symptoms showed beneficial behaviour regarding alcohol misuse and binge drinking, but only for a duration of up to 2 years. In contrast, adolescents suffering from anxiety or depression did not respond differently from the control group [17]. The same study showed that the intervention delayed the age of first alcohol use [17].

Most studies concluded that socioeconomic status (SES) did not play a significant role, with the exception of one study [16], which found that high-risk adolescents from higher SES with higher consumption and harm responded equally to those from lower SES. Furthermore, although high-risk adolescents exposed to peer victimisation had higher levels of problematic alcohol use (binge drinking, alcohol use, and alcohol-related harm) at the start of the intervention, the program demonstrated significant effects in reducing alcohol-related harm among them compared to their non-victimised peers [16]. A potential indirect effect was noted among low-risk peers, though the underlying mechanisms behind this outcome remain unclear [15]. Students having lower levels of education appear to benefit more from the intervention than students in higher education, regardless of gender [19].

#### 3.3.2. Unplugged

At three months follow-up, two studies reported an estimated 30% effect on use of cigarettes, cannabis, and alcohol, with significant reductions to the prevalence of daily cigarette use, episodes of drunkenness, and cannabis use [24,28]. Results were attributed to three common mediating factors: attitudes, refusal skills, and perception of prevalence of the behaviour among peers, while role-playing components were more impactful for students with prior substance use experience [28]. Short-term exposure also delayed progression towards daily cigarette smoking but did not reduce frequent use [24].

At fifteen months follow-up, the reduction in prevalence of cannabis use (26% estimated reduction) and episodes of drunkenness (38% estimated reduction) remained significant, yet there was no significant reduction in tobacco use despite the program’s impact on preventing progression towards initial use among non-users [25]. One study reported that participation resulted in significantly lower self-reported alcohol-related behavioural problems, including fighting, hospitalisations and accidents (22%) among students who did not drink at baseline and those who perceived that their parents tolerate drinking [23]. The same study also reported decreased likelihood to increase alcohol consumption compared to control; however, the overall frequency of alcohol consumption was not affected by participation in the program [23]. Similarly, in Slovakia, participation in Unplugged increased the likelihood of being a non-user of tobacco and alcohol and reduced the risk of becoming a new user [21]. The two Czech studies reported reduced smoking, heavy smoking, and cannabis use throughout two years post-intervention, but no significant effect on alcohol use, drunkenness frequency, and lifetime drug use [27,30]. In addition, another study reported preventative impacts on the speed at which cigarette use increased [26].

#### 3.3.3. IPSYcare

In terms of the pilot study [11], it was found that both teacher- and peer-led delivery methods were well received by students, but teacher-led sessions produced clearer reductions in alcohol and tobacco use than peer-led sessions. Peer-led sessions, however, were rated as more enjoyable and relatable.

One study found that during the three years of implementation, students who followed the IPSYcare program showed a smaller increase in monthly alcohol use compared with those in schools following the usual curriculum, although this difference was no longer evident two years after the program had ended [10]. Another study reported a consistent effect on cigarette smoking, with intervention participants maintaining lower smoking frequency over the 4.5-year follow-up [8,10].

Following the previously mentioned growth model, students that were classed into the normative (low-risk for problematic substance misuse) group had the most benefit from the IPSYcare program as it helped them delay the onset of drinking as well as the quantity of alcohol consumption. For the students identified in the problematic trajectory path, the program did not have a meaningful effect on reducing drinking frequency, with those who had already initiated drinking early on continuing along this path despite program participation [12]. For illicit drugs, measured as proneness to cannabis and ecstasy, IPSYcare students reported fewer intentions to experiment with and use such substances than their non-intervention peers, with the difference becoming greater at the final follow-up [8].

Earlier analyses conducted during the program implementation reported that IPSYcare students showed greater resistance to peer pressure, stronger school bonding, and lower levels of alcohol and tobacco use compared with controls [9]. In addition, improvements in school bonding were shown to partially mediate reductions in alcohol misuse [10]. Subgroup analyses also indicated that both school tracks benefitted from the program but in different ways, with students in the academic track showing larger improvements in knowledge about substances and skills for expressing themselves confidently in group settings, while those in the vocational track showed greater gains in resisting peer pressure and refusing substance offers [9]. Another subgroup analysis did not find gender to be a moderating factor when it came to the program’s effectiveness on alcohol-related behaviours but did find that gender moderated certain life skill outcomes, with girls showing greater gains in self-confidence and in understanding how to behave appropriately and assertively in group situations, whereas boys only showed minor improvements when it came to communication skills [9].

**Table 1:** Program characteristics and overall effectiveness.

Program	Characteristics	Effectiveness
Preventure	- Use of cognitive behaviour and personality profiling (AS, NT, IMP, SS) - Two 90 min sessions on personality traits knowledge and identification of problematic behaviours - 6 independent studies in the UK and Netherlands (2013–2019)	- Mixed results: binge drinking delay and reduced frequency. Not significant across all studies - Effective substance misuse prevention in high-risk individuals but mixed results on depression and anxiety - SES nor peer victimisation impact program effectiveness
Unplugged	- Based on the Comprehensive Social Influence model - Twelve 1 h weekly sessions to increase knowledge, build social skills, and foster interpersonal skills - 10 studies, six of them based on the EU-Dap trial (2004), three based on a Czech Republic study and one based on a Slovakian study	- 30% short-term reduction in cigarette, alcohol and cannabis use due to attitude shifts, refusal skills, and peer influence - Delayed progression to daily cigarette use, but no effect on frequency - Long-term reduction in cannabis and alcohol use but limited impact on tobacco
IPSYcare	- Use of youth behaviour psychological theories and life skills education model - Three-year intervention with student-professor role-plays and discussions - 5 studies (1 pilot study and 4 based on a 2003 quasi-experimental study in Germany)	- Decreased alcohol and tobacco consumption due to increased peer pressure resistance and school bonding - Effects most pronounced during intervention, with effects decreasing slightly two years after - Reduced students’ intentions to experiment with illicit substances

## 4. Discussion

The program characteristics and effectiveness are described in Table 1 (above). The findings indicate differences in the overall effectiveness between the programs, as well as in their relative impact on preventing or reducing alcohol, tobacco, and drug use. Additionally, their influence on behavioural outcomes varied. Preventure yielded mixed results with some evidence that it reduced binge drinking frequency in high-risk and non-high-risk individuals. However, this was not evenly reported across all studies, particularly among high-risk individuals with depression or anxiety. Most studies found no significant effect from socioeconomic status or peer pressure. Unplugged yielded a short-term reduction in cigarette, alcohol and cannabis use and was effective at delaying cannabis and alcohol use. However, its overall impact on cigarette smoking was limited despite delaying daily cigarette use. Lastly, IPSYcare reported decreased alcohol and tobacco consumption—with stronger effects during the three-year intervention period—and reduced illicit drug use over time.

### 4.1. Temporal Effects

Unplugged appears to have a protective effect, slowing progression and reducing the prevalence of problematic behaviours rather than preventing use [21,22,23,25]. Similarly, IPSYcare appears to reduce cigarette smoking frequency and substance use over time [9,10,31]. However, there was a minimal to no effect on alcohol consumption following program completion [8,27,30], suggesting that targeted interventions that incorporate the high-risk factors from Preventure could enhance the positive effect and prevention across all substances. In addition, program effectiveness was higher when combined with prevention strategies that had a family and social youth context dynamic [8].

### 4.2. Mediating Factors

Unplugged appears to reduce the intensity of cigarette smoking [24]. Adolescents with high self-esteem, home support, school connectedness, and prosocial peers were less likely to use alcohol and tobacco than their peers [21]. The program also decreased adolescents’ positive views of alcohol, cigarettes, cannabis, and other illicit drugs [21,28]. This connection may point to the continued usefulness of school-based prevention programs that increase adolescents’ inter- and intrapersonal skills [23,25]. In addition, while overall substance use increased between baseline and follow-up, the effect may be tied to the fact that use often begins during adolescence. This is consistent with three studies noting the limited impact on alcohol consumption due to the social acceptability of heavy drinking in social contexts [23,25,27].

Nevertheless, Unplugged has shown strong positive results and is considered as one of the most effective programs in Europe [24,25,29]. While one study conducted a systematic review concluding that Unplugged should be recommended, it is important to note that it only reviewed programs implemented in Spain, excluding Preventure and IPSYcare [29].

### 4.3. Relationship Between Effectiveness and Program Characteristics

The high level of Unplugged standardisation across its curriculum allows for uniformity of delivery and comparability of results; however, it reduces opportunities to include the local context [23]. Three studies were conducted in the Czech Republic and updated the program to shortened lessons, changed order, and added ice-breaker activities and graphics [26,27,30]. However, findings about whether the program was more effective were mixed. One study reported that the positive outcomes might have been influenced by the lack of other interventions [30]; whereas, another one reported that the lack of school prevention workers masks any potential improvements [27]. While IPSYcare provided one-day standardised training, no information was provided on if and how the program was adapted to different contexts.

IPSYcare and Unplugged incorporated role-play activities and group discussions involving students and teachers. IPSYcare increased its school bonding and participant responsiveness to the material, which increased program success [10]. Unplugged increased substance knowledge, mitigated positive attitudes towards substances, and addressed normative beliefs by practising decision-making and assertiveness. Benefits were more evident in students who had already experimented with substances, probably due to students having greater motivation to acquire resistance skills [28]. However, this has not been demonstrated in other studies and should be explored further.

Despite Unplugged being fully delivered in 56% of schools during the EU-Dap trial, significant reductions in drunkenness and cannabis prevalence were still reported fifteen months post-intervention, demonstrating the strengths of the program’s core components [21]. In addition, the delivery of the full program did not appear to have larger impacts, suggesting that even partial exposure has a strong influence on adolescents’ behaviours. Preventure and Unplugged shorter durations, can provide valuable insights into short-term outcomes [20], and the sustained reinforcement in IPSYcare contributes to the program’s sustainability [9,10].

### 4.4. Target Groups and Mechanism of Behavioural Change

Unplugged studies reported that interventions targeted at younger adolescents might be more impactful [25,26,27,30]. Similarly, the mean age of the students enrolled in IPSYcare was 10.5 years old, which provided the ideal timing for intervention before they start experimenting with substances, typically at 12 and 13 years old [8]. In contrast, Preventure targeted older adolescents identified as high-risk to address the underlying causes common to both alcohol use and mental health problems [17]. As a result, this is believed to have a dual effect, addressing both issues simultaneously [20]. While tailoring the program to the needs of at-risk groups increases the effectiveness of the program, it may miss opportunities for earlier prevention [26,27].

Preventure has a strong adaptability to different SES backgrounds, high-risk adolescents, or peer victimisation, resulting in the same benefits [16,19]. This highlights its potential for universal effectiveness. However, it is important to note that the identification of adolescents based on specific risk characteristics before intervention may limit the program’s generalisability to populations who don’t exhibit these traits but could still benefit. In contrast, the IPSYcare program targets a more diverse demographic, making it more effective for regular school curricula integration.

The herd effect is a particularly interesting mechanism among young people in school settings. The study by Conrod et al. (2013) highlighted the indirect influence that such programs can have on students [15]. These indirect effects suggest that behavioural changes among a subgroup of adolescents may influence the attitudes of the broader student population. It would therefore be relevant to further explore this phenomenon by including a social network analysis perspective. This would make it possible to identify key elements (i.e., group leaders or communication channels among students, such as social media applications) in order to better understand the mechanisms of diffusion through which preventive behaviours spread within peer groups. Moreover, this approach would allow for the comparison of social norms across different populations and the refinement of program interventions accordingly.

### 4.5. Strengths and Limitations

This review provides a comprehensive overview that can guide policymakers in their prevention strategies. All programs demonstrate, at least, partial effectiveness in addressing one or more substance risk behaviours. Further, it appears relevant to propose interventions tailored to students’ characteristics (e.g., Preventure), while ensuring that these interventions are sustainable in the long term (e.g., IPSYcare and its booster sessions) and scalable for larger implementations (e.g., Unplugged).

Although some studies were tested on a large and diverse population (e.g., EU-Dap trial), many of them were in fact secondary analyses based on the same initial data. Consequently, only six distinct baseline samples were examined, two for Preventure, one for IPSYcare, and three for Unplugged, limiting the ability to generalise these findings to other populations or contexts. At least some generalisability to the European context was demonstrated by the EU-Dap trial due to the multiple sociocultural contexts. Moreover, the results of each study should be interpreted within their demographic and cultural context, as it plays a key role in the intervention’s success. For instance, in the Netherlands, there is more liberal psychoactive substance use, which may influence baseline behaviours [16]. Another recent study conducted in Brazil’s initial Preventure program implementation yielded inclusive results; however, after adaptation to the local context (renamed #Tamojunto 2.0), effectiveness was reported [32].

Future research should directly compare school-based and community-based interventions in order to assess their complementary roles. Combining universal school programs with family or community-level strategies could enhance protective effects in the long term, extending impact well beyond a classroom setting. Evidence from the IPSYcare program illustrates this need, as IPSYcare was shown to be most effective for students in the normative development path but not so much for those who already displayed risky behaviours at baseline [12].

Among the studies, there was a scarcity in the number of qualitative studies. The findings presented above were primarily based on quantitative data, which focuses on measurable outcomes as opposed to subjective experiences. Qualitative data would provide in-depth insights and experiences from participants, as well as contextual factors. Importantly, because some studies reported a decline in program effectiveness following the implementation phase. However, a recent study conducted in Australia demonstrated the effectiveness of the Preventure program up to seven years post-intervention, providing confidence in the effectiveness of these programs and their long-term impact [31].

Although several studies used reliable and confidential self-reports to avoid biases such as peer desirability, other studies suggest that it might be worthwhile to explore alternative data collection methods in the future [10,18,19,20].

Another limitation of this review was the decision to exclude books and book chapters. This decision was largely taken due to time and resource constraints, which is a common challenge in scoping reviews that retrieve large numbers of publications. As highlighted in established methodological guidance, reviewers often have to balance the comprehensiveness of their search with what is feasible to complete [33,34]. While this approach may have excluded some useful contextual information, it helped maintain focus on studies with empirical data and comparable standards of quality. Future reviews could expand the scope to include books, chapters, or other grey literature in order to capture broader theoretical or program development insights.

## 5. Conclusions

This scoping review has identified and analysed the scope, reach, and impacts of three of the main substance abuse school-based prevention programs in Europe. The findings of this review reveal that while each of the programs show effectiveness in reducing alcohol and substance use among adolescent groups, their efficacy, particularly in the long term, varies significantly due to implementational and cultural differences. Preventure would benefit from tailoring its approach to address adolescents from different risk profiles. IPSYcare could extend its engagement duration and booster sessions to enhance impact. Unplugged may benefit from a more localised approach and continuous curricular updates in order to continue relevance and effectiveness. This review further identifies the need for more longitudinal studies that incorporate qualitative methods as well as the incorporation of familial/community engagement aspects into future preventions interventions that could help in the long run. Strengthening these aspects of the programs is vital in addressing substance abuse problematic behaviours—such as social and relational issues, binge drinking, hospitalisations, and fighting. Nevertheless, school-based interventions are essential tools in contributing to healthier communities and reducing burdens on public health systems in the long term.

## Data Availability

No new data were created or analysed in this study.

## Appendix A

**Table A1:** Data charting table including a description of articles on European school-based substance abuse prevention programs: programs, country, scope, and outcomes.

Title	Authors	Program	Country	Scope	Gender	Age	Changes acc to Context	Outcomes	Anything Interesting
Resilience factors, the school-based universal prevention program ‘Unplugged’ and healthy behavior among early adolescents	Abrinkova, L., Orosova, O., Neves De Jesus, S., Gajdosova, B., Bacikova-Sleskova, M. [21].	Unplugged	Slovakia	Alcohol, Tobacco	Total population: 425 participants (242 in the experimental group); 204 males and 221 females.	7th-grade students (mean age = 13.5)	The Unplugged program was implemented by teachers who completed a three-day training course. The implementation did not follow a strict time plan but depended on the availability and resources of each school individually.	Unplugged participation increased the likelihood of being a non-user and reduced the risk of becoming a new user (Unplugged has a protective effect against early-onset substance abuse).	Community environment is not associated with substance abuse among young adolescents. Higher self-esteem was significantly associated with a higher probability of being a non-user early adolescent. Being a female increased the probability of being a non-user adolescent when compared to males.
A Systematic Review of School-Based Alcohol and Other Drug Prevention Programs	Roberta Agabio, Giuseppina Trincas, Francesca Floris, Gioia Mura, Federica Sancassiani, Matthias C. Angermeyer [22].	Unplugged, SUCCESS, Adventure, CHOICE, Internet-based intervention, Media-based, P.A.T.H.S	Study 1—7 European countries, Study 2—Czech Republic, Study 3—7 European countries	Unplugged study 1—Alcohol, Alcohol related problems. 2. Unplugged study 2—Alcohol, Tobacco, Cannabis. 3. Unplugged study 3—Alcohol and other drugs	Study 1: Total population (7079): Females—3504 (49%); Males—3575 (51%). Study 2: Total population (1753): Females—859 (49%); Males—894 (51%). Study 3: Total population (6370): Females—3058 (48%); Males—3312 (52%)	Study 1: Ages 12–14 years; Study 2: Ages 11–13 years; Study 3: Mean age = 13.2 years.	Not reported	In one study, participation in Unplugged (compared with a control group that received usual health education) had a decreased risk of reporting alcohol-related problems. Moreover, non-drinkers and occasional drinkers progressed towards frequent drinking less. In study 3 there was a reduction (although marginal) in daily smoking, frequent smoking, and frequent cannabis use when compared to the control.	In Europe, the Unplugged program was the most evidence-based substance use prevention program
Effectiveness of a Selective, Personality-Targeted Prevention Program for Adolescent Alcohol Use and Misuse: A Cluster Randomized Controlled Trial	Conrod P.J., O’Leary-Barrett M., Newton N., Topper L., Castellanos-Ryan N., MacKie C. [15].	Preventure	United Kingdom	Alcohol	Total population: 2643 participants; High-risk (HR)—1210; Low-risk (LR)—1433.	Year 9 students (mean age = 13.7 years); follow-ups at 6, 12, 18, and 24 months.	Not reported	Significant reductions in rates of drinking, frequency of binge drinking, and problem drinking symptoms among high-risk adolescents. Indirect ‘herd effects’ were observed among low-risk adolescents as well as reduced binge drinking rates.	
The effect of contextual risk factors on the effectiveness of brief personality-targeted interventions for adolescent alcohol use and misuse: A cluster-randomized trial	Edalati, H., Afzali, M. H., Castellanos-Ryan, N., & Conrod, P. J. [16].	Preventure	United Kingdom	Alcohol	Intent to treat (final) pop: 1129		Not reported	Higher socioeconomic status (SES) adolescents had higher initial levels of alcohol use and related harm. However, they responded to the intervention just as effectively as those from lower SES and equally benefited from it.	Personality-targeted interventions proved effective for at-risk adolescents, even when peer victimisation went unnoticed by teachers or parents, highlighting a protective effect for this vulnerable group.
Sex specific trajectories in cigarette smoking behaviours among students participating in the Unplugged school-based randomized control trial for substance use prevention	Gabrhelik, R., Duncan, A., Lee, M. H., Stastna, L., Furr-Holden, C. D. M., & Miovsky, M. [26].	Unplugged	Czech Republic	Tobacco	Baseline: 944 males and 929 females (total population = 1874). Final follow-up: 906 males and 855 females (total population = 1871).	Baseline: Mean age = 11.8 years. Final follow-up: Mean age = 14.5 years.	Not reported	Useful for slow escalators independently of sex, not useful for rapid to moderate escalators independently of sex.	Identified two types of adolescent tobacco smokers: slow escalators and rapid moderate escalators. Distinguish between them, as the speed of progression is an indicator of nicotine addiction and heavy smoking progression.
Short-Term Mediating Factors of a School-Based Intervention to Prevent Youth Substance Use in Europe	Fabrizia Giannotta, Federica Vigna-Taglianti, Maria Rosaria Galanti, Maria Scatigna, Fabrizio Faggiano [28].	Unplugged	Austria, Belgium, Germany, Greece, Italy, Spain, and Sweden	Alcohol, Tobacco, Cannabis	Pre-test: 7079 students; Post-test: 6370 students (3057 females and 3312 males).	Mean age 13.25 years old	Not reported	Reduced cigarrete smoking, drunkness, and cannabis use.	Similarities in the mediating mechanisms for tobacco, alcohol, and cannabis use.
Prevention versus pseudo-prevention: A systematic review of school drug prevention and its indexing in best practice portals	Jorge Medina Martinez, Víctor José Villanueva-Blasco [29].	Unplugged	Spain	Alcohol, Tobacco, Cannabis, Marijuana	-	School age	Not reported	Unplugged efficient and recommended	
The effect of the school-based unplugged preventive intervention on tobacco use in the Czech Republic	Miovsky, M., Novak, P., Stastna, L., Gabrhelik, R., Jurystova, L., & Vopravil, J. [30].	Unplugged	Czech Republic	Tobacco	1874 Students: 952 males 922 females	Mean age 11.8 years old	Not reported	Unplugged is useful for slower progression to increased smoking but not statistically significant in the long term.	
The impact of youth internalising and externalising symptom severity on the effectiveness of brief personality-targeted interventions for substance misuse: A cluster randomised trial	Perrier-Ménard, Eveline;Castellanos-Ryan, Natalie;O’Leary-Barrett, Maeve;Girard, Alain;Conrod, Patricia J. [17].	Preventure	United kningdom	Alcohol	1025 students: 553 males 472 females	Mean age 13.7 years old	Not reported	Prevention is effective in reducing substance misuse in a two-year period in youth at particularly high risk for addiction and mental health problems.	
Translation of Etiology into Evidence-Based Prevention: The Life Skills Program IPSY	Karina Weichold [9].	IPSY	Germany (replication in Italy and Austria)	Alcohol and Tobacco	Pilot test: 105 participants (including pre- and post-test design; no gender specified). Large-scale study: approximately 1700 participants (no gender specified). The program was also tested on subgroups that included gender, but specific numbers were not provided in the review.	Ages 10.5–13 years (Grades 5 to 7)	Pilot test: the training manual has been revised slightly, mostly in a matter of time lines (based on the feedbacks of the teachers)	Outcome of pilot test: Need of a teacher-based implementation Outcome Large scale: IPSY seemed to have a positive effect on the prevalence, frequency of use and amount of use of alcohol and cigarettes. It’s effective to prevent early and high use of both substances. Compared to the control group, students who have followed the intervention program have had their general life skills and substance-specific competencies (e.g., resistance skills) increased. Both girls and boys profited from a positive effect on school bonding, general life skills and substance use. Girls have demonstrated a positive effect on communication competencies (effective communication and knowledge about self-confidence), but not boys. In this way, IPSY enables girls to strengthen their self-confidence and confidence in social interactions, which tend to diminish during early and middle adolescence. Outcome in the replication (Italy and Austria): IPSY targets universal risk and protective factors such as low adherence to social norms, influence from peers engaging in risky behaviours, or weak connections to structured developmental environments. IPSY can be effectively implemented in any cultural context.	687 students were non-white, but there is no analysis to differentiate the effects on white and Black populations.
Long-Term Effects of the Life Skills Program IPSY on Substance Use: Results of a 4.5-Year Longitudinal Study	Karina Weichold, Anja Blumenthal [8].	IPSY	Germany	Alcohol, tobacco and illicit substances	Gender not specified (all results did not change when controlling for the gender variable) Differents timing corresponding to the follow-ups: T1: 1657 T2: 1419 T3: 1278 T4: 1131 T5: 1014 T6: 685	Ages 10.5–15 years (Grades 5 to 7, with follow-ups in Grades 8 and 9).	not reported	Significant long-term effect on frequency of smoking cigarettes (less frequently) and proneness to cannabis and ecstasy use (less prone). Positive effect on alcohol consumption but only for a short term; during the 2 years following the end of the programme intervention, it starts to decrease again.	Even though it didn’t help for the alcohol consumption, the results show that participants of the intervention group drink alcohol not as much as a coping mechanism as the control group. In addition, later onset of alcohol use reduces the risk of addiction during adulthood (=lifetime benefit of the intervention). The high consumption of alcohol in Germany is part of their highly normative behaviour in mid-adolescence, when drugs and cigarettes are less accepted in the society for young people. The effectiveness of this program seemed to be higher when combined with prevention strategies involving families or other social contexts of youth.
The life skills program IPSY: Positive influences on school bonding and prevention of substance misuse	Victoria Wenzel, Karina Weichold, Rainer K. Silbereisen [10].	IPSY	Germany	Alcohol	N = 952 (54% female, 46% male)—number of students who completed both pre- and post-tests, as well as the follow-ups in Grades 6 and 7. Attrition was mainly due to schools withdrawing from the intervention for logistical reasons, or students changing schools or being absent due to illness.	Ages 10.5–13 years (Grades 5 to 7)	Alcohol use was measured at 4 intervals (pre-test=t1, post-test=t2, and then at the end of year 6=t3 and year 7=t4). Students were first asked to report their general alcohol consumption frequency over the previous 30 days using a scale ranging from 0/5 (0 being never and 5 being daily). Students were then asked to specify their previous alcohol consumption in the last 30 days for various types of beverages (beer, wine, mixed drinks, and spirits) as well as their future expectations regarding alcohol use within the next 12 months. In this study, although the school is not an explicit target of an LSP, the role of the bond that the school provides in the effectiveness of an LSP is analysed to determine whether it has an effect on alcohol misuse among young people	IPSY has a positive long-term effect on antecedents such as the intention to consume alcohol and on their actual consumption rate of alcohol. The positive influences on school bonding following program participation were found to partially mediate the intervention effects on alcohol use. Participating in this program reinforced their bond with school, which in return participated in a lower alcohol misuse behaviour.	IPSY has good timing in terms of implementation because it starts before the substance use is likely to occur (approx. between 12 and 14 in Germany) and before regular consumption patterns develop. The authors explained that IPSY’s declining impact on alcohol use is likely due to cultural factors in Germany surrounding alcohol’s social acceptability during mid-adolescence, making it difficult for school-based prevention alone to maintain effects beyond early adolescence. In contrast, tobacco and illicit drugs remain less socially accepted, which may explain IPSY’s longer-lasting protective outcomes. Students who completed IPSY also showed lower coping motives for drinking in that they were less likely to report using alcohol to manage stress or negative feelings, suggesting that IPSY had enduring effects on the psychological reasons for drinking even after use levels equalised with controls. Later booster sessions or integrating it with family- or community-based strategies were recommended by authors to sustain alcohol-related prevention gains over time.
Peers and Teachers as Facilitators of the Life Skills Program IPSY—Results from a Pilot Study	Karina Weichold, Rainer K. Silbereisen [11].	IPSY	Germany	Alcohol and Tobacco	Pilot study: 105 students (46 female, 59 male)	Ages 10.74–13 years (Grades 5 to 7)	The program was delivered either by teachers or by older peers (11th graders). Teacher-led implementation showed high fidelity (90% of manual content delivered), while peer-led delivery had lower fidelity (80%) and discipline issues during substance-related sessions.	Teacher-led IPSY: Later initiation and lower increase in alcohol use; reduced expectations of regular smoking; higher resistance to cigarette offers. Peer-led IPSY: Ineffective for alcohol and smoking outcomes; in some cases increased alcohol consumption and lowered resistance to cigarette offers. Both groups showed the normative increase in substance use with age, but only the teacher-led condition showed positive intervention effects.	Student satisfaction was notably higher in the peer-led interventions, with students rating the sessions 4.72/5 versus 4.04/5 in the teacher-led group (*p* < 0.01), and 100% wanted it offered again (vs. 88% in teacher-led). This shows that peers were perceived as more relatable and engaging, even though their sessions were less effective. The paper suggests that peers’ popularity and social closeness made them appealing facilitators but potentially undermined prevention goals, as all peer leaders drank alcohol and most had tried smoking. The authors recommend that peers be used for non–substance-related life skills sessions, while teachers lead the prevention-specific content. Facilitator credibility and attitudes toward substance use were highlighted as key moderators of program success.
Examining the Differential Effectiveness of a Life Skills Program (IPSY) on Alcohol Use Trajectories in Early Adolescence	Michael Spaeth, Karina Weichold, Rainer K. Silbereisen, Margit Wiesner [12].	IPSY	Germany	Alcohol and Tobacco	1484 students (47% male, 53% female)	Mean age at baseline = 10.5 years (Grade 5), with follow-up through Grade 7 (approximately age 13)	IPSY was implemented by trained teachers across 23 schools in Thuringia, Germany. Schools were randomly assigned to either the IPSY intervention or a control condition. The program ran for three years: 15 sessions in Grade 5, followed by 7 booster sessions in Grades 6 and 7. Teachers received annual one-day trainings before each implementation phase. Fidelity checks showed that teachers delivered around 80% of the planned material and achieved lesson goals in most sessions, suggesting good implementation quality. The program was embedded into normal school hours and applied in both urban and rural settings.	Researchers measured students’ alcohol use at three time points across Grades 5 to 7, recording both whether they drank and how much they drank. They used growth mixture modelling (GMM), a statistical method that groups individuals based on similar patterns of change over time. This analysis showed that not all adolescents followed the same developmental path in their alcohol use. Two main trajectories were identified. The first was a normative group, which included about 80 percent of students who either did not drink or showed a slow, typical increase in occasional drinking as they got older. The second was a problematic group, which made up about 20 per cent of students who began drinking earlier and showed a much steeper increase in both frequency and quantity of alcohol consumption. Compared with students in control schools, those who participated in IPSY were less likely to belong to the problematic group. Within the normative group, IPSY participants also showed a slower increase in alcohol use over time, meaning that the program helped delay and reduce drinking among the majority of students. These effects were described as small to medium in size, indicating a meaningful though moderate impact. However, IPSY did not significantly reduce drinking among students in the problematic trajectory. Those who were already drinking early or heavily continued on a high-risk path despite participation in the program. Overall, the findings suggest that IPSY was effective for general prevention but less suitable for adolescents who had already initiated alcohol use.	IPSY worked best for the majority of students following a typical developmental path, helping to slow the rise in alcohol use and keep consumption at lower levels. However, it was not effective for adolescents already showing risky behaviour at the start—suggesting that universal programs like IPSY need to be complemented by more focused interventions for high-risk youth. The study also showed that teacher training and fidelity were key to its success: consistent implementation in regular classes helped achieve stable outcomes.
Effectiveness of a selective intervention program targeting personality risk factors for alcohol misuse among young adolescents: Results of a cluster randomized controlled trial	Lammers, Jeroen;Goossens, Ferry;Conrod, Patricia;Engels, Rutger;Wiers, Reinout W.;Kleinjan, Marloes [18].	Preventure	Netherlands	Alcohol	699 (gender not specified)	13–15 years old	not reported	The outcome depends on the analysis strategy used: Logistic regression: no significant effects on binge drinking, alcohol use and problem drinking, or alcohol and binge-drinking frequency at 1-year post-intervention (snapshot). Latent growth: over the 12 months after the intervention, there’s significantly less growth in binge drinking and binge drinking frequency (delay). Use assessment: Alcohol Problem: based on RAPI, how often one has experienced each of 18 alcohol-related problems Alcohol use: binomial variable 0=none, 1 or more = 1 (in the past four weeks) Binge drinking: how many times one has had 5 or more drinks on one occasion during the past four weeks (binomial variable) Binge drinking frequency: same as binge drinking Alcohol use frequency: how often one has drunk in the past four weeks (ranging from 0 to 40)	LGC provides a more nuanced and useful perspective for understanding the effect of the intervention over time.
Effectiveness of a selective intervention program targeting personality risk factors: Results of interaction analyses	Lammers, Jeroen;Goossens, Ferry;Conrod, Patricia;Engels, Rutger;Wiers, Reinout W.;Kleinjan, Marloes [19].	Preventure	Netherlands	Alcohol	699 (gender not specified) No significant moderation effect was found for gender.	13–15 years old	not reported	The aim of the study was to analyse if certain theory-based subgroups benefit more from the programme than others. Young people having high sensation-seeking benefit from it when we talk about binge drinking and binge drinking frequency with a post hoc latent growth analysis. The ones having high anxiety sensitivity benefit from it when we look at the outcome of alcohol use 12 months post-intervention. No significant effect has been found for the personality traits negative thinking and impulsivity. No significant effect was found for the problem drinking measure. Regression analyses have been made for the primary analysis, and then latent growth has been made as post hoc analyses. Use assessment: Alcohol Problem: based on RAPI, how often one has experienced each of 18 alcohol-related problems Alcohol use: binomial variable 0=none, 1 or more = 1 (in the past four weeks) Binge drinking: how many times one has had 5 or more drinks on one occasion during the past four weeks (binomial variable) Binge drinking frequency: same as binge drinking Alcohol use frequency: how often one has drunk in the past four weeks (ranging from 0 to 40).	Selective prevention program, such as Preventure, seem to be more effective in modifying alcohol misuse behaviour among high-risk adolescents.
Effectiveness of a brief school-based intervention on depression, anxiety, hyperactivity, and delinquency: A cluster randomized controlled trial	Goossens, Ferry X.;Lammers, J.;Onrust, S. A.;Conrod, P. J.;de Castro, B. Orobio;Monshouwer, K. [20].	Preventure	Netherlands	Mental health outcomes such as depression, anxiety, hyperactivity, and delinquency	699 students; 48.5% female	Mean age = 14 years	not reported	The findings showed no significant overall effects of the intervention on these outcomes for the total high-risk sample. However, a significant positive effect was observed on anxiety reduction within the anxiety sensitivity (AS) personality group at 12 months post-intervention, while a negative effect on depression was noted in the negative thinking (NT) group.	
“Unplugged”: A school-based randomized control trial to prevent and reduce adolescent substance use in the Czech Republic	Gabrhelik, Roman;Duncan, Alexandra;Miovsky, Michal;Furr-Holden, C. Debra M.;Stastna, Lenka;Jurystova, Lucie [27].	Unplugged	Czech Republic	Alcohol, tobacco, drugs (cannabis)	1753 students; almost all male	Students in the 6th grade, mean age 11–13 y.o.	changed lesson order, graphics, shortened lessons, and included ice-breaker activities	This program effectively reduced smoking, heavy smoking, and cannabis use among primary school students in the Czech Republic, with statistically significant effects observed for these behaviours at the final follow-up. However, no significant intervention effects were found for alcohol use, frequent drunkenness, or lifetime drug use. The program demonstrated promising outcomes for tobacco and marijuana prevention, highlighting its potential as a universal prevention strategy for adolescent substance use in school settings.	
Effects of a School-Based Prevention Program on European Adolescents’ Patterns of Alcohol Use	Maria Paola Caria, Fabrizio Faggiano, Rino Bellocco, Maria Rosaria Galanti, [23].	Unplugged	7 European countries (Austria, Belgium, Germany, Greece, Italy, Spain, Sweden)	Alcohol (program also covers tobacco and illict drugs but study only reports on alcohol)	7079 students (49% of those that completed the follow-up were female)	12–14 years old	3-day interactive training course for teachers used to standardise implementation across countries.	Participation did not modify the risk of being a current drinker but did significantly lower self-reported alcohol-related behavioural problems by an estimated 22% (absolute risk reduction = 1.9%) among students who did not drink at baseline and those who perceived their parents to tolerate drinking; non-drinkers at baseline were more likely to remain non-drinkers and less likely to start drinking frequently, and occasional drinkers were less likely to become frequent drinkers.	Greater impact on alcohol-related problems than use frequency but did appear to slow the transitions between stages of drinking (nondrinkers more likely to remain nondrinkers, occasional drinkers show slower progression towards frequent drinking); Stronger effect on alcohol-related problem behaviours among boys.
The effectiveness of a school-based substance abuse prevention program: EU-Dap cluster randomised controlled trial	Fabrizio Faggiano, Maria Rosaria Galanti, Karl Bohrn, Gregor Burkhart, Federica Vigna-Taglianti, Luca Cuomo, Leila Fabiani, Massimiliano Panella, Tatiana Perez, Roberta Siliquini, Peer van der Kreeft, Maro Vassara, Gudrun Wiborg, [24].	Unplugged	7 European countries (Austria, Belgium, Germany, Greece, Italy, Spain, Sweden)	Alcohol, tobacco, drugs (cannabis)	7079 students (1497 female)	12–14 years old	3-day interactive training course for teachers used to standardise implementation across countries	Short-term exposure to the programme is linked to an estimated 30% effect on the use of cigarettes, cannabis, and alcohol (among the most effective school-based programmes), with significantly lower prevalence of daily cigarette use, episodes of drunkenness, and cannabis use in a 30-day window; it delays daily smoking rather than reducing frequent use.	Intervention arms that included parents or peer leaders did not appear more effective than the basic model; results could not be compared across countries due to statistical power and the short window of follow-up (under 4 months).
The effectiveness of a school-based substance abuse prevention program: 18-Month follow-up of the EU-Dap cluster randomized controlled trial	Fabrizio Faggiano, Federica Vigna-Taglianti, Gregor Burkhart, Karl Bohrn, Luca Cuomo, Dario Gregori, Massimiliano Panella, Maria Scatigna, Roberta Siliquini, Laura Varona, Peer van der Kreeft, Maro Vassara, Gudrun Wiborg, Maria Rosaria Galanti, [25].	Unplugged	7 European countries (Austria, Belgium, Germany, Greece, Italy, Spain, Sweden)	Alcohol, tobacco, drugs (cannabis)	7079 (47.8% of those who completed the 18-month follow-up were female)	12–14 years old	3-day interactive training course for teachers used to standardise implementation across countries	15 months post-exposure, we see significantly lower prevalence of cannabis use (26% estimated reduction) and episodes of drunkenness (38% estimated reduction) but no significant reduction in tobacco use despite preventing progression towards initial use.	The second follow-up to the study above showed similar results for cannabis and alcohol use but not for tobacco use, with similar issues with intervention arms.
